# Malignancy dominated with rheumatic manifestations: A retrospective single-center analysis

**DOI:** 10.1038/s41598-018-20167-w

**Published:** 2018-01-29

**Authors:** Jian Wen, Han Ouyang, Ru Yang, Lin Bo, Yi Zhang, Mei Tang, Zhichun Liu

**Affiliations:** 1 Department of Rheumatology and Immunology, The Second Affiliated Hospital of Soochow University, Suzhou, 215004 China; 2 Department of Nephrology, The Second Affiliated Hospital of Soochow University, Suzhou, 215004 China

## Abstract

Paraneoplastic rheumatic syndromes comprise a heterogeneous group of disorders characterized by typical rheumatic manifestations but without direct invasion by the tumor or metastases. The clinical features and malignancy-associated risk factors of 21 patients with paraneoplastic rheumatic syndromes, including 11 men and 10 women with a mean age of 56.3 ± 13.1 years, were characterized by a retrospective review. All patients were diagnosed with malignancy within 2 years of rheumatism diagnosis. Patients suffering from solid malignancies accounted for the majority (62%); hematological malignancies were observed in the remainder. Arthritis (48%), lymph node enlargement (38%), skin rash (38%), weight loss (29%), fever/chills (24%), fatigue (24%), muscle soreness (24%) and smoking history (29%) were common findings. Except for 8 patients (38%) who tested positive for anti-nuclear antibody (ANA) and 9 positive for rheumatoid factor (RF), all patients tested negative for anti-extractable nuclear antigen (ENA) antibodies. Rheumatic disorders with a typical clinical presentation in older patients and nonspecific systemic features should alert clinicians to search for an occult malignancy. Patients with rheumatic disease must be closely followed to screen for malignancies, particularly within 2 years of rheumatism diagnosis.

## Introduction

Paraneoplastic syndromes involve symptoms mediated by hormones and cytokines from a tumor or are the consequence of humoral or cellular immune mechanisms directed against tumor cells, though direct invasion by the tumor or metastases does not occur. Several studies have indicated connections between malignancy and rheumatic manifestations^1–3^. In the case of paraneoplastic rheumatic syndromes, rheumatic symptoms can coincide, precede, or follow the diagnosis of cancer or herald its recurrence^4^, generally at no longer than 2 years before the diagnosis of associated cancer^5–7^. Malignancies are associated with a wide variety of paraneoplastic rheumatic manifestations, which may arise in joints, fasciae, muscles, vessels or bones^8,9^. However, most paraneoplastic rheumatic syndromes are difficult to distinguish from idiopathic rheumatic disorders; thus, cancer occurrence may constitute a major diagnostic challenge. Early detection and therapy may be of utmost clinical importance. Therefore, this study aimed to examine the relationship between rheumatic manifestations and malignancy to further improve our understanding of paraneoplastic rheumatic disease and to avoid misdiagnosis.

## Methods

### Data Sources

A total of 21 patients with paraneoplastic rheumatic disease in longitudinal medical care at the Second Affiliated Hospital of Soochow University between January 2011 and January 2016 were included in this study. The group consisted of the following: six rheumatoid arthritis (RA) cases; four Sjogren’s syndrome (SS) cases; four vasculitis cases; two undifferentiated connective tissue disorder (UCTD) cases; and one case each of polymyositis (PM), ankylosing spondylitis (AS), adult-onset still’s disease (AOSD), systemic lupus erythematosus (SLE) and polymyalgia rheumatica (PMR). None of the patients had a history (including personal and family history) of cancer.

The diagnostic criteria for this study included the classification standard of the American College of Rheumatology for RA and SLE, New York criteria for AS, 2012 revised International Chapel Hill Consensus Conference classification criteria for vasculitis, 2002 International Classification Criteria for SS, Bohan and Peter criteria for PM, Doran criteria for PMR, Yamaguchi’s criteria for AOSD and Corte and colleagues’ criteria for UCTD.

The 21 patients with paraneoplastic rheumatic disease were divided into three groups according to the dominant clinical manifestation: arthritis (n = 7), vasculitis (n = 4) and other connective tissue disorder (CTD) (n = 10) groups. The CTD group was composed of four SS cases, two UCTD cases and one case each of PM, AOSD, SLE and PMR. The data obtained were compared among the three groups.

The patients’ medical records, including symptoms, physical findings, smoking status and history of cancer, were thoroughly reviewed. Laboratory findings such as anti-nuclear antibody (ANA), anti-extractable nuclear antigen (anti-ENA) antibody, rheumatoid factor (RF), anti-cyclic citrullinated peptide (anti-CCP), and anti-neutrophil cytoplasmic antibody (ANCA) were evaluated at the time of diagnosis. All patients had sufficient pathological examination and radiological investigation data to confirm the diagnosis of malignancy. The study methods were approved by the Ethics Committee Board of the Second Affiliated Hospital of Soochow University (Approval No. LK2016032), and all methods were performed in accordance with relevant guidelines and regulations.

### Statistical analysis

All statistical analyses were performed using the GraphPad Prism (version 4.0) statistical program. Continuous variables are expressed as the mean ± standard deviation (SD).

## Results

### General conditions of the patients with paraneoplastic rheumatic disease

The characteristics of all patients are summarized in Table 1. A total of 21 patients (11 men and 10 women) with paraneoplastic rheumatic disease, including one fatal case, were included in this study. The mean age at diagnosis of paraneoplastic rheumatic disease was 56.3 ± 13.1 years. In total, 29% of patients with paraneoplastic rheumatic disease were current smokers. The mean age of onset for patients with arthritis manifestation was 62.4 ± 14.2 years, which was older than that of patients with vasculitis (57.5 ± 6.5 years) or other CTDs (51.6 ± 13.4 years). All four patients presenting with vasculitis were males, and one patient died.

**Table 1:** General conditions of the patients with paraneoplastic rheumatic disease.

		Total	Arthritis	Vasculitis	Other CTDs
Subjects		21 (100)	7 (33)	4 (19)	10 (48)
Age at diagnosis (years)		56.3 ± 13.1	62.4 ± 14.2	57.5 ± 6.5	51.6 ± 13.4
Male		11 (52)	4 (57)	4 (100)	3 (30)
Alive		20 (95)	7 (100)	3 (75)	10 (100)
Dead		1 (5)	0	1 (25)	0
Smoking Status	Current	6 (29)	3 (43)	1 (25)	2 (20)
	Former or never	15 (71)	4 (57)	3 (75)	8 (80)

Data are presented as mean ± SD or n (%).

### Clinical manifestations of the patients with paraneoplastic rheumatic disease

Arthritis (48%), lymph node enlargement (38%), skin rash (38%), weight loss (29%), fever/chills (24%), fatigue (24%) and muscle soreness (24%) were common findings at the time of paraneoplastic rheumatic disease diagnosis. In addition, skin ulcer (19%) and limb numbness (14%) were relatively common complaints (Table 2).

**Table 2:** Clinical manifestations of the patients with paraneoplastic rheumatic disease.

Symptoms	All patients N = 21	Arthritis N = 7	Vasculitis N = 4	Other CTDs N = 10
Fever/chills	24%	29%	50%	10%
Fatigue	24%	0	50%	30%
Weight loss	29%	14%	50%	30%
Lymph node enlargement	38%	57%	25%	30%
Skin rash	38%	0	100%	40%
Subcutaneous nodule	5%	0	25%	0
Skin ulcer	19%	0	50%	20%
Arthritis	48%	100%	0	30%
Symmetric polyarthritis	33%	71%	0	20%
Muscle soreness	24%	0	50%	30%
Numbness of limb	14%	0	50%	10%
Headache	5%	0	25%	0
Hearing lose	5%	0	25%	0

Values are presented as n or %.

The patients in the arthritis group tended to manifest with symmetric polyarthritis (71%), lymph node enlargement (57%) and fever (29%). Two of the four vasculitis patients with malignancy presented with fever, weight loss, acute symmetrical myalgia, skin ulcer, and fatigue.

### Laboratory parameters in paraneoplastic rheumatic disease

Autoantibody testing was performed in all patients (Table 3). Eight patients (38%) were positive for ANA, but all tested negative for anti-ENA antibody. RF positivity was found in nine patients (43%). Two patients were positive for anti-CCP antibodies (41 AU/mL and 85 AU/mL) and two for ANCA (Table 3).

**Table 3:** Autoantibody profile in patients with paraneoplastic rheumatic disease.

Autoantibody	All patients N = 21	Arthritis N = 7	Vasculitis N = 4	Other CTDs N = 10
ANA	38%	14%	25%	60%
Anti-dsDNA	0	0	0	0
Anti-nucleosome	0	0	0	0
Anti-nonhistone	0	0	0	0
Anti-Sm	0	0	0	0
Anti-U1RNP	0	0	0	0
Anti-Ribosomes-P protein	0	0	0	0
Anti-SSA	0	0	0	0
Ro-52	0	0	0	0
Anti-SSB	0	0	0	0
Anticentromere	0	0	0	0
Anti-Scl70	0	0	0	0
Anti-Jo-1	0	0	0	0
RF	43%	57%	25%	40%
anti-CCP	10%	29%	0	0
ANCA	10%	0	50%	0

ANA, anti-nuclear antibody. RF, rheumatoid factor. anti-CCP, anti-cyclic citrullinated peptide. ANCA, anti-neutrophil cytoplasmic antibody. Values are presented as n or %.

### Rheumatic disease and malignancy

All of the 21 patients with paraneoplastic rheumatic manifestation developed malignancy within 24 months of rheumatism diagnosis (Table 4); the mean time interval between rheumatic manifestations and malignancy was 8.0 ± 7.7 months. Nineteen patients were diagnosed with malignancy after rheumatism diagnosis (average, 8.9 ± 7.5 months postdiagnosis), one before rheumatism diagnosis (2 months pre-diagnosis), and one coincident with rheumatism diagnosis. All four cases of patients with suspected vasculitis developed malignancy within 2–4 months. Three patients were diagnosed with lymphoma within 18 months after SS diagnosis, and the other was diagnosed 2 months before SS.

**Table 4:** Diagnosis and type of malignancy in patients with paraneoplastic rheumatic disease.

Patient number	Sex	Malignancy diagnosis delay(months)	Rheumatism diagnosis	Type of malignancy	Malignancy detection method
1	M	12	RA	Renal Clear Cell Cancer	Biopsy
2	F	3	AOSD	Gastric antrum adenocarcinoma	Biopsy
3	M	2	Vasculitis	Metastatic adenocarcinoma	Biopsy
4	M	24	PM	Multiple myeloma	Bone arrow morphology
5	F	0	RA	Peripheral T-cell lymphoma	Biopsy
6	F	12	RA	Breast cancers	CT scan
7	F	1	RA	Peripheral T-cell lymphoma	Biopsy
8	M	4	Vasculitis	Lung adenocarcinoma	Percutaneous lung puncture biopsy
9	M	2	PAN	Liver cancer	CT scan
10	M	3	GPA	Lung adenocarcinoma	Sputum cytology
11	M	24	RA	Ascending colon carcinoma	Biopsy
12	F	12	UCTD	Breast cancers	Biopsy
13	F	13	SLE	Breast cancers	Biopsy
14	F	-2	SS	NHL	Biopsy
15	M	9	AS	Lung squamous carcinoma	Biopsy
16	F	13	SS	NHL	Biopsy
17	F	18	SS	NHL(B-cell lymphoma)	Biopsy
18	F	12	SS	NHL	Biopsy
19	M	1	RA	Prostate Cancer	MRI
20	M	3	PMR	Prostate Cancer	Biopsy
21	M	1	UCTD	Peripheral T-cell lymphoma	Biopsy

RA, Rheumatoid arthritis. AOSD, adult-onset still’s diseases. PM, polymyositis. PAN, polyarteritis nodosa. GPA, granulomatosis with polyangitis. UCTD, undifferentiated connective tissue disease. SLE, systemic lupus erythematosus. SS, Sjogren’s syndrome. AS, Ankylosing spondylitis. PMR, polymyalgia rheumatica. NHL, Non-Hodgkin’s lymphoma. CT, Computed Tomography. MRI, Magnetic Resonance Imaging.

Among the 21 cases of paraneoplastic rheumatic disease, 18 were confirmed via pathology results, and the remaining patients were confirmed via computed tomography (CT) or magnetic resonance imaging (MRI) scan. Solid malignant tumors (n = 13), including lung cancer (n = 3), breast cancer (n = 3), liver cancer (n = 2), prostate cancer (n = 2), gastric cancer (n = 1), colon cancer (n = 1), and renal cell carcinoma (n = 1), accounted for the majority (62%) of diagnosed cancers. A total of 71% of patients with suspected arthritis and 100% of those with suspected vasculitis developed solid tumors. In addition, hematological malignancies accounted for 8 cases (38%), namely, 7 cases of lymphoma and 1 case of multiple myeloma. In our study, all four patients with suspected SS ultimately developed lymphoma.

## Discussion

Since the first case of paraneoplastic rheumatic disease was reported in 1916, the number of cases in the literature has increased annually. An incidence of paraneoplastic rheumatic disease of 2.65%–23.1% has been reported, and all types of rheumatism or rheumatic symptoms are present in malignant diseases^10–13^. Previous studies have also shown that malignancy typically occurs within 24 months in patients with paraneoplastic rheumatic disease^14,15^. In the present study, all patients with paraneoplastic rheumatic manifestation developed malignancy within 24 months of rheumatism diagnosis, with the majority (76%) developing malignancy within 12 months.

Based on our results, 71% of our patients presented with polyarthritis, which corroborates the findings of previous studies^16–18^. These patients often present with significant alterations in their general condition, fever, and sudden onset of an unusual inflammatory arthritis. Although the pathogenesis of paraneoplastic polyarthritis remains unclear, immune mechanisms involving mediators, such as hormones, cytokines, peptides, antibodies, and cytotoxic lymphocytes, have been implicated^19^. We also observed a high percentage of male patients (57%; 4/7), with a mean age of 62.4 ± 14.2 years. As RA disease frequently occured in women, we speculate that such a late age of onset in male patients may be an important feature of cancer polyarthritis. Moreover, several features of malignancy-related polyarthritis, as defined by Zupancic *et al*.^19^, were also observed in this study, including the absence of deformities, rheumatoid nodules, and previous family history of rheumatoid arthritis. Similar to current research showing that RF is increased in 23–42% of patients with malignancy^17,20^, 43% of patients with malignancy tested positive for RF in our study. Additionally, two patients were positive for anti-CCP antibodies. As anti-CCP has been reported to have a high specificity (90–96%) for RA^21,22^, this may demonstrate that using positive results of RF and anti-CCP antibody to distinguish between paraneoplastic rheumatic syndromes and RA are not always reliable. Nonetheless, higher titers of RF and anti-CCP were mostly found in early-onset RA patients^20^.

This study included four cases of vasculitis with malignant tumors, accompanied by fever, weight loss, acute symmetrical myalgia, skin ulcer and fatigue. In addition, lymph node enlargement, subcutaneous nodule, skin rash, hearing loss, and headache were also noted, similar to other studies^23,24^. The patients were all males, and their ages ranged from 51–64 years old. Two patients with vasculitis were positive for ANCA. These results suggest that the diagnosis of vasculitis must be made with caution, as ANCA testing may also be positive in malignancy. A few proposed mechanisms of vasculitis as a paraneoplastic syndrome include immune complex formation (persistent antigen stimulation from the tumor stimulates T cell activation), direct vascular injury by antibodies to endothelial cells, and a direct effect of leukemic cells on the endothelium^25,26^.

The types of malignancy in ANCA-associated vasculitis (AAV) patients also differ. Several studies have reported that hematological malignancies, such as lymphoma^27^ and leukemia^28^, are common, and solid tumors in the bladder and lungs have also been reported^27^. However, the association between vasculitis and tumor type cannot be confirmed through statistical analysis because of the limited number of cases. In this study, all four vasculitis patients were diagnosed with solid tumors: two cases of lung adenocarcinoma and two cases of liver cancer. The results indicate that all suspected vasculitis patients had malignancy at 2–4 months after vasculitis diagnosis, which suggests that monthly screening of malignancy, particularly solid tumors, is needed within 6 months following a diagnosis of vasculitis.

In other CTD patients with malignancy, we observed one case of AOSD, which manifested as fever, arthritis, loss of appetite, sore throat, and an extensive red rash mostly on the face, trunk, and arms. The patient was ultimately diagnosed with gastric antrum adenocarcinoma after 3 months. The dermatitis was bright red, even flaming or brownish red, particularly on the face and head, with branch expansion of the capillaries. This dermatitis is termed malignant erythema or a variant of dermatitis; it has a poor prognosis and is highly suggestive of malignancy. Therefore, when malignant erythema occurs and treatment with glucocorticoid is insufficient, potential tumors should be considered, even if no tumor is found. These patients should be comprehensively examined for cancer and provided with long-term follow-up.

One case of PM reported in our study developed malignancy after 24 months. However, current research shows that the greatest risk for malignancy exists within the first year following the diagnosis of PM and dermatomyositis (DM)^29,30^, which may suggest that when the diagnosis of PM is established, determining cancer risk is necessary, and further diagnostic assessments are indispensable. Wide and symmetrical muscle atrophy, disproportionate muscle strength and muscle atrophy, and acute necrotizing myopathy may suggest malignancy. Although some studies have reported that PM is strongly associated with certain types of malignant lymphomas, particularly Hodgkin’s lymphoma^31,32^, our result indicates that one patient manifesting as PM developed multiple myeloma(MM), which is in accordance with the result reported by Jowitt SN *et al*.^33^. These findings may indicate that PM is more related to hematological malignancies.

SS has already been reported as a risk factor for non-Hodgkin lymphoma (NHL), likely in relationship with a persistent stimulation of B cells during its course. In this study, four patients were diagnosed with both SS and NHL, none of whom were positive for specific autoantibodies such as RF, anti-SSA or anti-SSB antibody. In addition, short time intervals between SS manifestations and lymphoma (average 10.2 months, range −2–18 months) were observed, differing from the clinical features of lymphoma that develop in primary SS (pSS), as the sera of patients with SS frequently contain positive RF and antinuclear antibodies. Furthermore, the risk of NHL development in SS increases with time, and a fivefold increased risk of NHL was found to persist for > 10 years of disease duration for both pSS and secondary SS^34^. Lymphoma can induce paraneoplastic manifestations such as neuropathy and vasculitis, and the same symptoms may also occur as extraglandular manifestations of pSS^35^. Differentiating between these two causes is difficult. Prospective research will be needed to more accurately delineate the relationship between disease manifestations and lymphoma.

In terms of SLE, several studies have reported the development of malignancy in association with SLE. In the present study, we found one SLE patient with breast infiltrating ductal carcinoma. A previous study that examined 180 breast cancers in an SLE cohort^36^ reported the most common histological type to be ductal carcinoma (n = 95.66%), followed by lobular adenocarcinoma (n = 11.8%).

In summary, paraneoplastic rheumatic disease is common and easily confused with rheumatic disease. Our retrospective review reveals that rheumatic disorders presenting with atypical clinical symptoms in older patients, nonspecific systemic features such as unexplained fever, fatigue, weight loss, lymph node enlargement and skin rash, and clinical findings compatible with well-recognized paraneoplastic syndromes should alert clinicians to the possible coexistence of an occult malignancy. Regular follow-up is essential for these patients, particularly within 2 years of rheumatism diagnosis, and monthly screening of tumors is needed within 6 months following diagnosis of vasculitis. Routine assessment and screening for malignancy, particularly solid tumors, in patients with suspected arthritis and vasculitis should be considered. Screening for lymphoma in patients with suspected SS should also be considered.
